# Editorial: Interplay Between Fungal Pathogens and Fruit Ripening

**DOI:** 10.3389/fpls.2020.00275

**Published:** 2020-03-24

**Authors:** Juan C. Díaz Ricci, Luis González-Candelas, Dov B. Prusky, Claudio Moser, Elena Baraldi

**Affiliations:** ^1^Facultad de Bioquímica, Química y Farmacia, Instituto Superior de Investigaciones Biológicas (INSIBIO, UNT-CONICET), San Miguel de Tucumán, Argentina; ^2^Department of Food Biotechnology, Institute of Agrochemistry and Food Technology (IATA), Paterna, Spain; ^3^Department of Postharvest Science, Agricultural Research Organization - the Volcani Center, Institute of Postharvest and Food Sciences, Rishon LeZion, Israel; ^4^Centro Ricerca e Innovazione, Fondazione Edmund Mach, San Michele all'Adige, Italy; ^5^Laboratory of Plant Pathology and Biotechnology, DISTAL- Alma Mater Studiorum University of Bologna, Bologna, Italy

**Keywords:** postharvest, fungal pathogens, ripening, plant defense, virulence, fruit

The fresh fruit industry relies on the capability of farmers and distributors to offer the best quality fruits to consumers, as consumers always choose fruits that look both attractive and delicious. However, if we want fruits to remain in a commercially good condition, preserving their appetizing and attractive appearance, and avoiding premature rot due to the infection of postharvest pathogens, it becomes mandatory to understand all possible aspects related to the mechanisms involved in such an unwanted and apparently unavoidable process. Using this conceptual frame, we envisioned the importance studying how postharvest pathogens attack fruits, how they grow, the pathogenic mechanism used, and the optimal or restrictive conditions provided by the fruit, and that which is required by microorganisms to exert their pathogenic effects.

These considerations are of particular relevance when a healthier and safer technology is required to lead horticultural production to more eco-friendly management. In addition, substituting synthetic fungicides by bioproducts requires knowing the mechanisms pathogens use in fruit rot, and to find possible targets to halt fruit invasion and pathogen growth. The latter turns out to be even more critical in berry fruits as they are consumed with their peals (i.e., strawberry, blueberry, cranberry, raspberry, etc.) or other fruits that may also be consumed with their peals (i.e., tomato, apple, pear, plum, peach, etc.).

With these ideas in mind, we proposed a Research Topic focused on the “Interplay between Fungal Pathogens and Fruit Ripening” with the aim to gather papers of researchers working in this specific issue to provide new information about this particular issue.

After evaluating the research papers sent by our potential contributors, we chose six that most closely and accurately fit the subject of our Research Topic. Research papers on the interaction between *Botrytis cinerea* with grapes, strawberry and tomato, *Fusarium acuminatum* with tomato, *Fusarium ananatum with* pineapple, *Colletotrichum acutatum* with strawberry, *Penicillium expansum* with apples, and *Rhizopus stolonifer* with tomato were selected and are briefly commented on below.

In the contribution Haile, Malacarne et al., the authors apply a dual approach to investigate the processes activated in *V. vinifera* fruit and in the pathogen *B. cinerea* during quiescence and egression fungal stages. Results obtained constitute a valuable contribution to the knowledge of the pathogenic mechanisms activated by the fungal pathogen *B. cinerea* in its attempt to invade grape fruits on one side, and, the defense response activated by the grapes to retaliate the pathogen attack on the other side.

In the paper Haile, Nagpala-De Guzman et al., the authors use a transcriptomic approach to investigate the changes of gene transcription levels occurring at different strawberry fruit ontogeny stages (e.g., open flowers, white and red fruits) when infected with the pathogen *B. cinerea*. They observed that unripe fruits activate a stronger defense response than completely mature fruits, and *B. cinerea* remains quiescent in white fruits.

In Barral et al., the authors apply an imaging analysis approach to study the evolution of the disease called “fruitlet core rot,” caused by the fungal pathogen *Fusarium ananatum*. Advanced imaging technologies such as X-ray, fluorescence, and multiphoton microscopy are applied to identify particular features displayed on pineapple fruit tissues when invaded by the pathogen. Different fruit tissues were analyzed, and the infection process was evaluated at different infection stages. Results obtained lead to the conclusion that anatomy changes and lignification are important defense mechanisms activated against the fruitlet core rot disease.

The contribution titled Petrasch et al. is a comparative study on the mechanisms utilized by three important fungal pathogens (e.g., *B. cinerea, F. acuminatum*, and *R. stolonifer*) to invade tomato fruits at different ripening stage. The idea is to test the hypothesis that the ripening stage is important to make tomato fruits more susceptible to the pathogens differentially activating the infection mechanisms of each pathogen. A transcriptome analysis is conducted to analyze pathogenic and virulence parameters in unripe and ripe tomato fruits. The authors conclude that the ripening stage of the fruit is important to allow the pathogens grow.

In the paper Higuera et al., the authors evaluate the effect of the expression, in strawberry fruits, of the transcription factor FaWRKY1 on the defense response against the hemibiotrophic fungal pathogen *C. acutatum*. Using Agrobacterium-mediated transient transformation to silence or overexpress the gene FaWRKY1 in strawberry fruits that are later infected with the fungal pathogen, authors report that the expression of FaWRKY1 exerts a negative effect on the induction of the defense response of strawberry fruits against *C. acutatum*.

Finally, the paper Kumar et al. evaluates the effect of the gene *laeA* and genes involved in the synthesis of patulin, on apples infected with the fungal pathogen Penicillium expansum. Different apple ripening parameters are evaluated as well as their effect on the expression of other associated *laeA* regulated genes, and on the accumulation of the mycotoxin patulin.

Results presented clearly unveil that there is a complex crosstalk between fruits and pathogens, and the switch from the quiescence to the active pathogen stage depends, among other things, on the fruit ripening stage. We hope that these contributions shed light on the complex mechanisms used by some fungal pathogens to infect the host fruits.

## Author Contributions

JD wrote the article. All other authors made intellectual and revision contribution, and approved for its publication.

### Conflict of Interest

The authors declare that the research was conducted in the absence of any commercial or financial relationships that could be construed as a potential conflict of interest.

